# Rank-Based Detection of Gravitational-Wave Transients Using Chatterjee Correlation

**DOI:** 10.3390/s26154662

**Published:** 2026-07-23

**Authors:** Daniel Beltran Martinez, Carlos Delgado Mendez, Carlos Diaz Ginzo, Pablo Garcia Abia, Salvatore Mangano, Gonzalo Merino, Gaia Volpi

**Affiliations:** 1 Centro de Investigaciones Energeticas, Medioambientales y Tecnologicas (CIEMAT), E-28040 Madrid, Spain; daniel.beltran@ciemat.es (D.B.M.); carlos.delgado@ciemat.es (C.D.M.); carlos.diaz@ciemat.es (C.D.G.); pablo.garcia@ciemat.es (P.G.A.); merino@pic.es (G.M.); 2 Port d’Informacio Cientifica (PIC), Campus UAB, E-08193 Bellaterra, Spain; 3 Dipartamento di Fisica e Astronomia “G. Galilei”, Università di Padova, I-35131 Padova, Italy; gaia.volpi@studenti.unipd.it

**Keywords:** gravitational waves, burst searches, transient detection, Chatterjee correlation, rank statistics, Advanced LIGO, data analysis

## Abstract

We present a rank-based method for detecting short-duration gravitational-wave transients in 46 days of coincident data from the first Advanced LIGO observing run (O1). The method applies a moving-window implementation of Chatterjee’s rank correlation coefficient to whitened interferometric sensor strain data. This produces a computationally efficient statistic sensitive to temporally ordered signal structure without relying on waveform templates. Compared with traditional excess power and coherent burst searches, the rank-based formulation is potentially less sensitive to certain non-Gaussian noise transients. Furthermore, it processes dual-detector data faster than real time on a single CPU core. We evaluate the method using 60 hardware injections from the O1 dataset, recovering 28 compact binary coalescence injections, primarily for events with a single-detector signal-to-noise ratio above approximately 13. The pipeline identifies 31 transient candidates, including the astrophysical event GW150914 and two instrumental glitches. Although the present implementation is less sensitive than established search pipelines, these results demonstrate the feasibility of the new method. Rank-based detection statistics provide a computationally efficient and complementary method for low-latency transient detection in interferometric sensor networks.

## 1. Introduction

Gravitational waves are transient deformations of spacetime produced by accelerating massive systems. They are observed by ground-based laser interferometers, such as Advanced LIGO, Virgo, and KAGRA [1,2,3,4], which operate as highly sensitive sensor systems measuring differential arm-length changes induced by spacetime strain. These instruments monitor such variations to capture signals from astrophysical sources, including binary black hole and neutron star mergers.

Traditional gravitational-wave searches rely primarily on template-based methods, such as matched filtering, which correlate detector data with large banks of modeled waveforms [5,6,7]. These techniques are highly sensitive to signals predicted by general relativity, particularly from compact binary coalescences (CBCs), in which black holes and neutron stars merge through their inspiral, merger, and ringdown phases. However, the sensitivity of template matching degrades when astrophysical signals deviate from available analytical or numerical models. To address this limitation, several morphology-independent methods have been developed to identify short-duration, unmodeled transient signals [8,9,10,11,12,13,14].

We develop a method to identify coincident excesses across multiple detectors without waveform templates. Our approach transforms strain data into a rank-based detection statistic derived from Chatterjee’s rank correlation coefficient [15]. This coefficient measures the dependence between signal amplitude and time within a moving window and is computed through a rank-based formulation with weak sensitivity to the underlying amplitude distribution. Unlike Spearman’s ρ or Kendall’s τ, which are primarily sensitive to monotone relationships, Chatterjee’s coefficient approaches unity for general functional dependence, potentially allowing sensitivity to a broader class of transient morphologies. To our knowledge, this is the first application of Chatterjee’s rank correlation coefficient as a detection statistic for gravitational-wave transient searches.

The statistic is evaluated in sliding windows across whitened strain data to produce a time series sensitive to local deviations from stationary Gaussian noise. Candidate events are identified by selecting statistically significant excesses and requiring temporal coincidence between detectors. In contrast to traditional excess power searches, this detection statistic is based on the rank-ordered correlation of signal value and time rather than energy. While the present implementation employs a single window length, the procedure can be generalized to a bank of window durations to provide sensitivity across multiple timescales. This procedure defines a computationally efficient pipeline for detecting short-duration, unmodeled gravitational-wave transients. Beyond gravitational-wave astronomy, transient detection in distributed sensor networks operating under non-Gaussian noise presents a related general problem. In this context, rank-based statistics provide an attractive, low-latency alternative to traditional amplitude-based methods due to their computational efficiency and robustness against outliers.

The present work describes the algorithmic development and initial performance characterization of this pipeline on data from the first Advanced LIGO observing run (O1). No background estimation is performed, and no astrophysical significance claims are made regarding the O1 dataset beyond the hardware injection efficiency results. These components are left to future work.

The structure of the paper is as follows. Section 2 describes the data selection and pre-processing steps. The algorithmic implementation is detailed in Section 3, and its performance evaluation is presented in Section 4.

## 2. Data Selection

The data selection process begins with the acquisition of coincident strain data from the O1 observing run. The data are then merged, resampled, and whitened, followed by automated quality checks to exclude periods with missing data. This procedure yields a cleaned dataset suitable for the subsequent rank-based correlation coefficient analysis described in Section 3.

### 2.1. Data Acquisition

We analyze publicly available strain data from O1, obtained through the Gravitational Wave Open Science Center (GWOSC) [16]. Data access and initial processing are performed using the GWpy software package [17], which provides standardized tools for handling gravitational-wave time series.

Coincident strain data from the Hanford and Livingston detectors are divided into 60 s segments, each padded by one second at both ends to reduce edge effects. For each segment, we require that both detectors provide valid data over the full duration, that both channels contain the same number of samples, and that no more than 5% of the samples in either detector are NaN. Segments failing any of these criteria are discarded. Valid segments are stored in a compact binary format, including detector identifiers, GPS timestamps, and the corresponding strain samples.

After applying these selection criteria, the final dataset contains 1106.4 h (46.1 days) of coincident data. For comparison, the O1 analysis reported in Ref. [18] quotes 48.6 days of coincident data after data quality selection.

### 2.2. Data Pre-Processing

The previously generated (see Section 2.1) adjacent 60 s segments are concatenated into continuous intervals of up to 20 min, removing redundant samples at overlaps. Each detector time series is then resampled to a uniform 4096 Hz grid, with short gaps filled using locally computed mean values. This procedure ensures a continuous standardized input for the search pipeline.

The merged data are whitened using a median-based power spectral density estimate computed from four-second Fast Fourier transforms with a Hann window and a two-second overlap. Prior to spectral estimation, a constant detrending is applied. To suppress low-frequency noise, a high-pass filter with a 30 Hz cutoff is applied during whitening. The whitening itself employs a frequency-domain inverse spectrum truncation method with a filter duration of two seconds. To minimize edge artifacts introduced by filtering and the whitening process, the first and last second of each processed segment are removed.

The resulting uniformly sampled, high-pass filtered, and whitened time series are saved in binary files. Due to the removal of edge artifacts after the whitening process, the total available time is slightly reduced. The final dataset contains a total of 1102.8 h (46.0 days) of usable data.

## 3. Algorithm

The pre-processed strain data from Section 2.2 serve as input to the transient search algorithm, which targets short-duration, unmodeled gravitational-wave signals. The method employs a rank-based detection statistic, providing reduced sensitivity to certain non-Gaussian noise fluctuations while avoiding reliance on specific waveform templates. For each detector, the strain time series is transformed into ranks and evaluated independently within a moving window to produce a continuous statistic time series. These per-detector statistics form the basis for subsequent coincidence analysis across the detector network. The implementation of this procedure is described below.

### 3.1. Rank-Based Correlation Coefficient Calculation

Classical measures of statistical association, such as Pearson’s correlation coefficient, quantify specific forms of dependence, most notably linear relationships. In applications involving unmodeled transient signals, where the underlying structure is not specified in advance, it can be advantageous to consider dependence measures that are sensitive to broader classes of relationships.

Chatterjee’s rank-based correlation coefficient [15] provides a rank-based nonparametric measure of dependence between two random variables. Defined entirely in terms of ranks, it is robust to outliers, invariant under monotone transformations, and supported by a simple asymptotic theory that enables efficient hypothesis testing without resampling. The coefficient takes values in the interval from 0 to one, where 0 indicates statistical independence and one corresponds to a deterministic functional relationship.

In its original formulation [15], the coefficient $\xi_{N}$ between the variables *X* and *Y*, with *Y* ranked by increasing *X*, is defined as

(1) $$\xi_{N}\left(X,Y\right)=1-\left(\frac{3}{N^{2}-1}\sum_{i=0}^{N-2}\left|R\left(t_{i}\right)-R\left(t_{i+1}\right)\right|\right),$$

where $R(t_{i})$ denotes the zero-based rank of the *i*-th sample. Under the null hypothesis of stationary Gaussian noise and in the limit of large *N*, $\xi_{N}$ is asymptotically normally distributed with mean 0 and variance 2/(5N).

To exploit these properties for gravitational-wave detection, we apply Chatterjee’s coefficient within a moving time window to quantify the dependence between the strain signal, playing the role of *Y*, and time, playing the role of *X*, in the equation above. In random noise, the coefficient remains close to 0, whereas it approaches unity if the strain varies smoothly across the window. This continuous, rank-based measure naturally serves as the detection statistic computed by our pipeline.

Building on this idea, the algorithm processes the whitened strain time series independently for each detector. Let

(2) $$h(t_{i}),\;i=0,1,\ldots ,N-1,$$

denote the *N* consecutive samples within a sliding window. Each strain value is replaced by its corresponding rank,

(3) $$R(t_{i}),\;i=0,1,\ldots ,N-1,$$

which represents the position of the value $h(t_{i})$ in the sorted set of windowed strain values. The smallest value is assigned rank 0 and the largest rank N−1. To ensure that every sample is assigned a unique rank, ties are resolved deterministically according to their insertion order within the sliding window. Specifically, when two strain samples have identical values, the sample entering the window most recently is assigned the higher rank. Although exact ties are rare for whitened strain data, this deterministic rule guarantees a unique and reproducible ordering of the window contents. For sufficiently large *N*, the value of the random variable $R(t_{i})/N$ is approximately uniformly distributed in the [0, 1) interval, independently of the original distribution of the strain values $h(t_{i})$.

For each window position *t*, we compute the rank difference statistic

(4) $$S_{N}\left(t\right)=\sum_{i=0}^{N-2}\left|R\left(t_{i}\right)-R\left(t_{i+1}\right)\right|,$$

where $R(t_{i})$ denotes the rank of the *i*-th sample within the current window. The statistic $S_{N}\left(t\right)$ has its minimum value when h(t) is a strictly monotonic function of time within the window, since consecutive samples then have nearly consecutive ranks. For irregular dependencies of the strain with *t*, including stochastic fluctuations, the rank differences increase, leading to larger values of $S_{N}\left(t\right)$. If the values of h(t) within the window are statistically independent of time ordering (i.e., they are independent and identically distributed), the ordering of ranks is effectively random. In this case, $S_{N}\left(t\right)$ follows a well-defined null distribution determined by *N*.

To obtain a dimensionless detection statistic, the rank difference statistic is normalized as

(5) $$S^{\prime }\left(t\right)=\left(1-\frac{3S_{N}\left(t\right)}{N^{2}-1}\right)\sqrt{2.5N}.$$

The quantity inside the parentheses is identical to Chatterjee’s rank correlation coefficient. Under the null hypothesis of independence, the asymptotic variance of this coefficient is 2/(5N) [15], and multiplication by the normalization factor $\sqrt{5N/2}=\sqrt{2.5N}$ produces a statistic with asymptotic unit variance.

Under the assumption of stationary Gaussian noise and in the limit of large *N*, the marginal distribution of the normalized rank difference statistic $S^{\prime }\left(t\right)$ approaches a Gaussian distribution with zero mean and unit variance. Significant deviations from this baseline indicate temporally ordered structure within the analysis window, which may arise from either astrophysical transients or instrumental noise fluctuations.

In practice, deviations from ideal conditions, due, for example, to finite sample size or non-stationary noise, lead to departures from the theoretical Gaussian normalization of $S^{\prime }\left(t\right)$. The pipeline therefore applies a segment-level renormalization to produce the final rank-based detection statistic S(t). For each processed data segment, the mean μ and standard deviation σ of $S^{\prime }\left(t\right)$ are computed over all time samples in that segment, and the renormalized rank-based detection statistic is defined as

(6) $$S\left(t\right)=(S^{\prime }\left(t\right)-\mu )/\sigma .$$

This step ensures that the threshold S(t)>5 corresponds approximately to a standardized 5σ excess across all segments.

### 3.2. Running Time

To evaluate the running time of the algorithm described above, we use a fixed window length of N=128 samples. For a sampling frequency of 4096 Hz, this corresponds to a time span of approximately 31 ms, which is well matched to the characteristic timescale of short gravitational-wave transients such as CBCs, whose merger phase lasts on the order of tens of milliseconds in the detector frequency band. Within this window, the rank-based detection statistic is primarily sensitive to locally ordered temporal structure in the strain amplitude.

This window slides across the data, and the internal rank ordering is maintained using an ordered ring buffer. The samples within the window are stored in a balanced ordered container. In this representation, both insertion and deletion operations require O(logN) time. As the window advances by one sample, only a single outgoing value is removed and a single incoming value is inserted. Because no full re-sorting is required, the computational cost per step is reduced from O(NlogN), as required for repeated sorting, to O(logN).

With this configuration, the implementation is computationally efficient, processing dual-detector data approximately 70 times faster than real time. For example, a 1200 s interval of coincident data is fully analyzed in about 33 s of wall-clock time. The reported timing results were obtained on an Intel Xeon Gold 6148 CPU (2.4 GHz) using a single processing core and include the stages of rank computation, normalization, and renormalization.

Although the present implementation employs a single fixed window length, the pipeline can be extended to a bank of analysis windows covering a broader range of transient durations. Assuming the same ordered data structure is used, the computational cost scales logarithmically with window size and approximately linearly with the number of window configurations. Increasing the window size from approximately 30 ms to 500 ms introduces only a negligible increase in the computational cost of an individual configuration. By contrast, a bank comprising five window lengths (32, 64, 128, 256, and 512 ms) would increase the overall execution time by approximately a factor of five while remaining compatible with low-latency processing. Such a multi-window strategy is expected to improve sensitivity to longer-duration transients. The principal trade-off is that larger analysis windows contain more detector noise, potentially reducing the statistical significance of short-duration signals, making threshold optimization across multiple timescales an important direction for future work.

### 3.3. Independent Detector Processing

The rank-based detection statistic is computed independently for the Hanford (H1) and Livingston (L1) detectors, generating two continuous time series. The results are stored in a structured data format optimized for efficient access and analysis [19]. Each entry includes a sample index, a GPS timestamp, the high-pass filtered and whitened strain data h(t), and the corresponding rank-based detection statistic S(t). This dataset enables direct comparison between the strain amplitude and the rank-based detection statistic during follow-up studies.

### 3.4. Excess Search

The excess search operates on the rank-based detection statistics $S_{H1}\left(t\right)$ (Hanford) and $S_{L1}\left(t\right)$ (Livingston), along with their corresponding timestamps.

Candidate intervals are defined through coincident threshold crossings. An interval is initiated when both statistics satisfy

(7) $$S_{H1}\left(t\right)>5\;\mathrm{and}\;S_{L1}\left(t\right)>5$$

at coincident sample times.

The threshold value of five corresponds to formally a one-sided Gaussian tail probability of $2.9\times 10^{-7}$ per independent sample under ideal stationary Gaussian noise. In practice, non-stationary noise transients and temporal correlations modify the effective false alarm rate (FAR), and this threshold is therefore treated as a heuristic trigger level rather than a formally calibrated detection threshold. A full background characterization via time-slides is deferred to future work.

The candidate interval is terminated as soon as one of the detectors falls below the threshold. This procedure yields a set of disjoint time intervals during which the joint detector response remains continuously above the threshold.

For each identified interval, all processed samples between the corresponding start and end indices are examined. Within this interval, the absolute maxima of the statistics are determined independently for the two detectors (H1 and L1) as follows:

(8) $$S_{H1}^{\max}=\max\left(S_{H1}\right)\;\mathrm{and}\;S_{L1}^{\max}=\max\left(S_{L1}\right).$$

To avoid multiple excesses from the same physical event, a fixed dead time of one second is applied. If two candidate excesses occur within this interval, only the larger excess is retained. The GPS timestamps at which these maxima occur are denoted $t_{H1}^{\max}$ and $t_{L1}^{\max}$. A representative event time is defined as the mean $t_{\mathrm{event}}=\frac{1}{2}\left(t_{H1}^{\max}+t_{L1}^{\max}\right)$. The temporal consistency between detectors is evaluated through the absolute time difference $\Delta t=\left|t_{H1}^{\max}-t_{L1}^{\max}\right|.$ The maximum gravitational-wave propagation delay between the Hanford and Livingston detectors is approximately 11 ms.

For every retained interval, the procedure records the detector maxima $S_{H1}^{\max}$, $S_{L1}^{\max}$, the event time $t_{\mathrm{event}}$, the time delay Δt, and a reference to the originating data segment. These quantities constitute the final output of the excess search and serve as input to the performance analyses described in Section 4.

**Figure 1:**
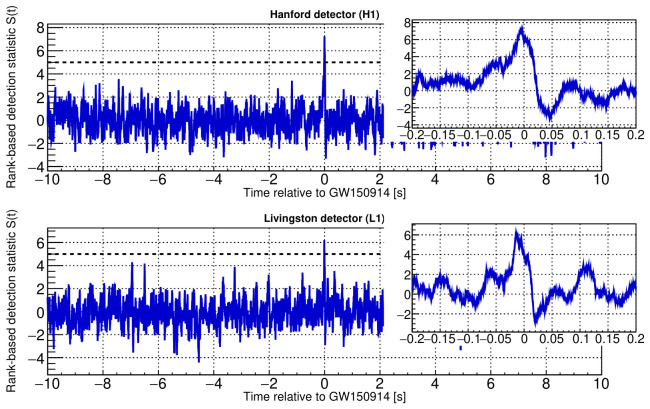
Rank-based detection statistic as a function of time for the Hanford (H1) and Livingston (L1) detectors around GW150914. The statistics $S_{H1}\left(t\right)$ (**upper panel**) and $S_{L1}\left(t\right)$ (**lower panel**) are evaluated continuously on whitened strain data. At the time of GW150914, both detectors exhibit peaks surpassing the threshold value of 5 (dotted line), indicating statistically significant coincident excesses. The inset panels show a zoomed view (±0.2 s) around the peak. The observed relative shift of 5.4 ms between detectors is consistent with the expected gravitational-wave propagation time.

An example of such a coincident excess is shown in Figure 1. The figure illustrates the response of the rank-based detection statistic in both detectors around the GW150914 event. A clear, coincident peak is observed at GPS time 1,126,259,462.4 s, with values $S_{H1}\left(t\right)\approx 7.3$ and $S_{L1}\left(t\right)\approx 6.2$, demonstrating the sensitivity of the method to a strong CBC signal.

## 4. Algorithm Performance Using Simulated Hardware Injections

During the O1 run, simulated gravitational-wave signals, known as hardware injections, were introduced into the detectors to validate the performance of search pipelines [20]. These injections were performed coherently in both detectors and comprised three waveform morphologies: CBCs, long-duration burst signals, and white-noise bursts.

Each injection is documented with parameters, such as GPS time, masses, source distance, waveform type, and expected and recovered signal-to-noise ratios (SNRs). This dataset provides a benchmark for assessing the performance of algorithms under realistic operating conditions.

To evaluate the performance of the pipeline, we compare the recovered excesses (Section 3.4) with the official catalog of simultaneous H1 and L1 hardware injections from the O1 run.

### 4.1. Matching Procedure Between Hardware Injections and Pipeline Excesses

Hardware injections and coincident pipeline excesses are matched using their GPS times. A pipeline excess is considered associated with a hardware injection ($t_{\mathrm{inj}}$) if the absolute time difference satisfies $\left|t_{\mathrm{inj}}-t_{\mathrm{event}}\right|<2\;s$. The relatively broad matching window is adopted to accommodate the simplified event-time assignment procedure presently used by the pipeline. All hardware injections and pipeline excess pairs that satisfy this condition are classified as *matched events*. Injections without a corresponding pipeline excess within this time window are labeled *missed injections*, while pipeline excesses without a nearby injection are classified as *unmatched excesses*.

Each pipeline excess is evaluated using an automated inspection tool. The pipeline extracts the relevant data segment and generates four time-series plots spanning a symmetric window of ±0.1 s centered on $t_{\mathrm{event}}$. The displayed quantities include the whitened strain amplitudes and the rank-based detection statistics for both the Hanford and Livingston detectors.

This visualization process enables direct inspection of detector behavior in the immediate vicinity of each excess. Comparing the structural features of the strain amplitude with the rank-based detection statistic provides a practical method to distinguish between short-duration noise transients, baseline threshold fluctuations, and genuine astrophysical signals.

**Figure A1:**
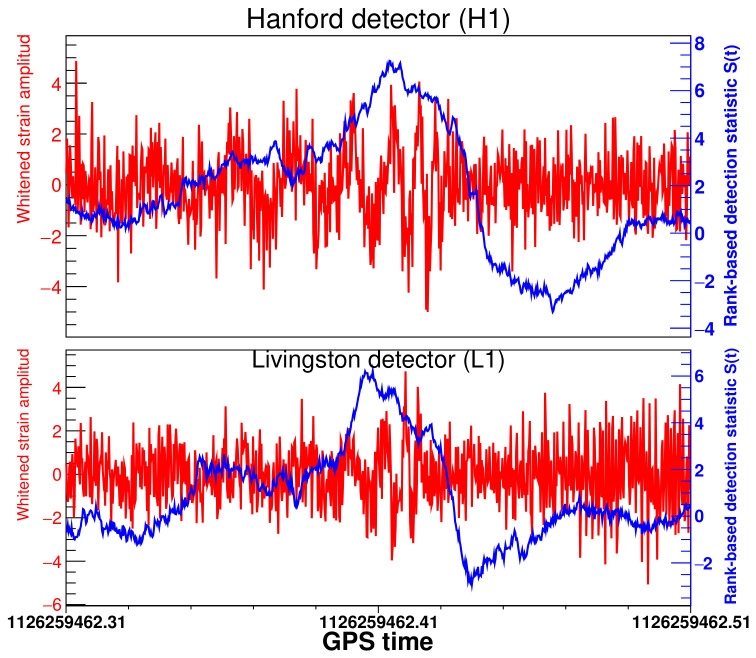
Time series of the whitened strain and rank-based detection statistic S(t) for the GW150914 event. The whitened strain (red, left axis) and the corresponding rank-based detection statistic S(t) (blue, right axis) are shown as a function of GPS time for the Hanford (**upper panel**) and Livingston (**lower panel**) detectors. The S(t) statistic exhibits a prominent, coincident enhancement in both detectors, demonstrating the pipeline’s sensitivity to short-duration gravitational-wave transients.

**Figure A2:**
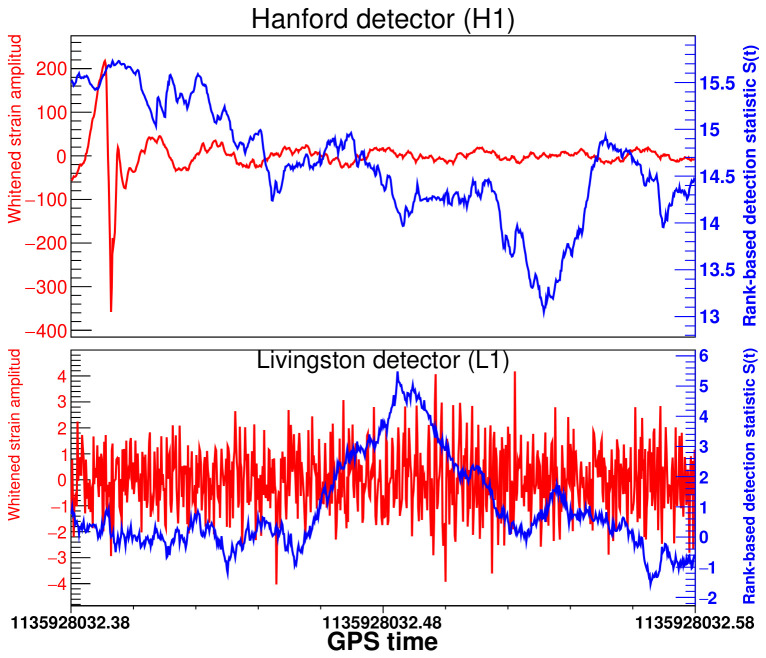
Time series of an instrumental noise transient during the O1 run. The whitened strain (red, left axis) and the corresponding rank-based detection statistic S(t) (blue, right axis) are shown as a function of GPS time for the Hanford (**upper panel**) and Livingston (**lower panel**) detectors. A large amplitude glitch in the Hanford detector, reaching a whitened strain amplitude of approximately 200, produces a coincident threshold crossing in the Livingston detector. The extreme strain amplitude and morphology are consistent with an instrumental artifact rather than an astrophysical signal.

**Figure A3:**
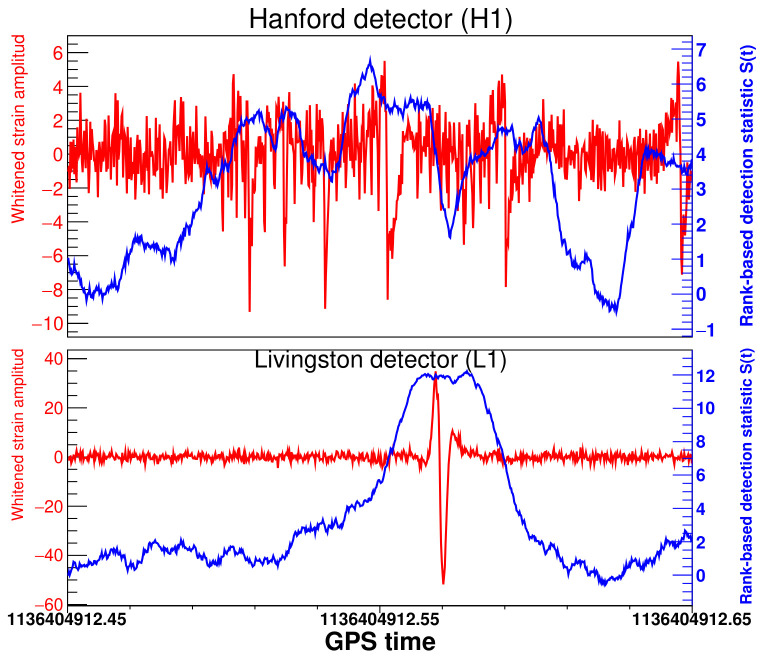
Time series of an instrumental noise transient during the O1 run. The whitened strain (red, left axis) and the corresponding rank-based detection statistic S(t) (blue, right axis) are shown as a function of GPS time for the Hanford (**upper panel**) and Livingston (**lower panel**) detectors. A glitch in the Livingston detector, reaching a whitened strain amplitude of approximately 40, produces a coincident threshold crossing in the Hanford detector. The event is consistent with an instrumental transient that would likely be rejected by standard data quality vetoes.

To illustrate the behavior of the pipeline in practice, representative examples of three unmatched excesses identified in the full O1 run are presented in Appendix A (Figure A1, Figure A2 and Figure A3). These include the astrophysical event GW150914, which is formally classified as unmatched here because it is absent from the hardware injection catalog, alongside two coincident instrumental glitches exhibiting pronounced, short-duration strain excursions. Together, these examples illustrate how the diagnostic time-series comparison distinguishes authentic gravitational-wave signals from loud noise transients that accidentally satisfy the network coincidence conditions.

### 4.2. Detection Efficiency

The detection efficiency of the pipeline was evaluated using 60 hardware injections simultaneously present in both detectors during O1. The sample comprises 54 CBCs, five long-duration burst injections, and one white-noise burst.

After applying the selection criteria described in Section 2 and Section 3, including the criteria detailed in Section 4.1, the pipeline identified 31 coincident excess events. Cross-matching these events with the hardware injection catalog shows that 28 are associated with injected CBC signals (classified as matched events). The remaining three excesses have no corresponding hardware injections and are therefore classified as unmatched excesses.

The recovered CBC signals correspond to the strongest injections, with the weakest detected event having an expected single-detector SNR of approximately 13. For nearly all these matched events, the time delay between detectors Δt is smaller than 11 ms, indicating accurate temporal localization by the pipeline. Only two matched events show slightly larger offsets of approximately 13 and 20 ms. These deviations are likely caused by the simplified procedure used to select the event maxima in the pipeline.

A total of 32 injected events were not recovered by the pipeline. Inspection of these missed injections indicates that they fall into three categories:

- **Low SNR CBC injections:** These events typically have an expected single-detector SNR between 3 and 13, producing rank-based detection statistics that are insufficient to surpass the threshold in both detectors.
- **Long-duration burst injections:** Their signal morphology extends beyond the fixed analysis window, meaning the signal is only partially captured by the 31 ms window size.
- **Broadband white-noise bursts:** These bursts lack the ordered temporal structure to which the rank-based method is most sensitive. This result is expected, as the pipeline is tailored for coherent transient signals rather than stochastic-like emissions.

Of the three unmatched excesses presented in Figure A1, Figure A2 and Figure A3 in Appendix A, one corresponds to the astrophysical event GW150914. The remaining two unmatched excesses exceed the coincidence threshold in both detectors but show no corresponding injection. Time-series inspection reveals that both events coincide with prominent short-duration glitches in the strain data, characterized by abnormally large strain values. These pipeline excesses are therefore interpreted as noise transients that briefly satisfy the coincidence condition.

Excluding GW150914, the search yielded two unmatched coincident excesses out of 31 pipeline excesses. In the absence of a dedicated background analysis, these events are interpreted as candidate accidental coincidences associated with instrumental noise transients.

**Figure 2:**
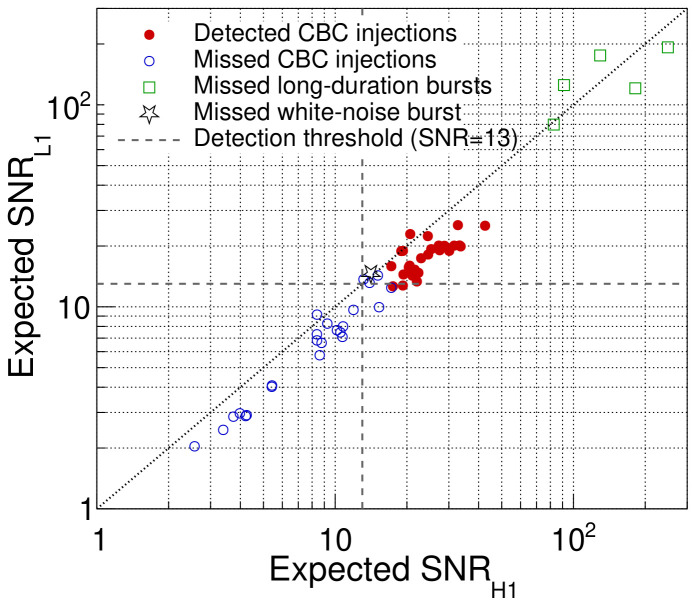
Expected single-detector SNR distribution for coherent hardware injections. Red and blue points represent recovered and missed injections, respectively. Marker styles distinguish between compact binary coalescences (CBCs), long-duration bursts, and stochastic white-noise burst injections. The dotted diagonal line indicates equal expected SNR in both detectors. The vertical and horizontal dashed lines indicate the SNR = 13 threshold, above which the pipeline recovers the majority of CBC injections.

Figure 2 summarizes the detection performance by showing the expected single-detector SNR distribution of all hardware injections, distinguishing between recovered and missed events. Recovered injections cluster at higher single-detector SNR values, while missed injections are predominantly concentrated at lower SNR or correspond to signal morphologies not captured by the algorithm.

Our method demonstrates high recovery efficiency for CBC injections with expected single-detector SNR ≳13, while sensitivity decreases rapidly below this level. By comparison, established gravitational-wave search pipelines, such as matched filtering for CBC searches and morphology-independent burst searches such as cWB [21], typically achieve high detection efficiency at SNRs of approximately eight. Rather than competing with these existing pipelines, the objective of this work is to demonstrate the feasibility of a computationally efficient, template-free detection statistic based on Chatterjee’s rank correlation coefficient. The recovery of 28 hardware injections, together with the detection of GW150914, provides an initial characterization of the pipeline’s performance and establishes a baseline for future improvements in detection sensitivity.

### 4.3. Cross-Correlation Analysis and Ongoing Developments

In addition to the single-detector rank-based detection statistic presented in Section 3, we investigate the incorporation of a cross-correlation analysis between the outputs of the two detectors as a complementary consistency test. This approach evaluates the degree of correlation between time-shifted data streams within the maximum gravitational-wave propagation time between detectors. By maximizing the correlation over short time segments selected by the rank-based detection statistic and applying appropriate pre-processing of the strain data, the cross-correlation may help to discriminate coherent transient signals from coincident noise fluctuations.

The time lag at which the maximum cross-correlation is achieved provides an estimate of the signal propagation time between the detectors, while its magnitude quantifies the degree of coherence, distinguishing astrophysical signals from uncorrelated background noise. In particular, we explore both Pearson cross-correlation and rank-based correlation estimators derived from the Chatterjee coefficient, to capture different types of statistical dependence between the detector outputs. The combined use of these estimators is expected to strengthen the identification of coherent signals while reducing the impact of spurious noise-induced coincidences.

As an illustrative example, among the three events presented in Appendix A, the GW150914 event (Figure A1) exhibits larger Pearson and Chatterjee correlation values than the two instrumental noise events shown in Figure A2 and Figure A3.

Furthermore, consistency tests comparing both the relative rank ordering and the absolute strain amplitude of candidate events between the two detectors can serve as complementary vetoes against accidental coincidences. Instrumental glitches often produce abnormally large or mutually inconsistent strain excursions between detectors, whereas genuine gravitational-wave signals are expected to exhibit amplitudes broadly consistent with the expected detector responses. Such consistency tests, analogous to those employed in coherent search pipelines, may further suppress coincident instrumental glitches, such as those illustrated in Figure A2 and Figure A3, while preserving sensitivity to coherent astrophysical signals.

Beyond these proposed consistency tests, further methodological developments are also under investigation, including the use of variable analysis window sizes to improve sensitivity to longer-duration transients, alternative statistical ordering schemes to enhance sensitivity to diverse signal morphologies, and more sophisticated coincidence metrics. In parallel, systematic threshold optimization is under investigation through improved discrimination strategies between signal and background. These approaches may be adapted to target specific classes of signals, for example in dedicated studies of highly eccentric compact binary systems or close encounters.

These extensions are expected to enhance sensitivity across a broader range of signal durations and morphologies. However, they also increase the effective trials factor and may increase the FAR. Ongoing studies therefore focus on quantifying this trade-off in order to maximize detection efficiency while maintaining controlled background levels.

## 5. Conclusions

We have presented a rank-based pipeline for detecting short-duration gravitational-wave transients. This framework applies Chatterjee’s rank correlation coefficient within an ordered sliding-window implementation, applied to Advanced LIGO O1 data for initial performance characterization.

The pipeline recovers 28 of 54 CBC hardware injections, showing substantial recovery efficiency for events with expected single-detector SNR ≳ 13, with decreasing sensitivity rapidly below this level. All five long-duration burst injections and the single white-noise burst injection are missed, indicating that extension to a multi-window analysis is likely the most impactful near-term improvement. The astrophysical event GW150914 is recovered with rank-based detection statistics $S_{H1}\approx 7.3$ and $S_{L1}\approx 6.2$, demonstrating sensitivity to an astrophysical CBC signal. Instrumental glitches caused two coincident excesses, corresponding to an observed unmatched excess rate of approximately 16 yr^−1^ in the analyzed dataset. No time-slide background analysis was performed, so this value does not represent a calibrated FAR. The observed unmatched excess rate is higher than the calibrated FAR values reported for established morphology-independent burst pipelines such as cWB [21] and BayesWave [22], which achieve FAR below the 0.01 yr^−1^ scale for significant events in O1. This difference reflects the absence of data quality vetoes and a dedicated background estimation in the present implementation.

The computational efficiency of the pipeline makes it naturally suited to low-latency applications, which is its primary potential advantage over more computationally intensive methods. Three developments are required before the pipeline can become a competitive search pipeline: (i) the implementation of a multi-window analysis employing window durations from approximately 30 ms to 500 ms to improve sensitivity to longer-duration transients; (ii) the incorporation of vetoes, including cross-correlation consistency tests between detectors, to reduce the FAR from ∼16 yr^−1^ to levels comparable with established pipelines; and (iii) a full background characterization to replace the heuristic threshold with a formally calibrated detection threshold. If these developments yield sufficiently low detection thresholds in SNR together with controlled FAR, the pipeline would represent a viable complement to existing burst searches in future observing runs.

## Data Availability

The data analyzed in this study are publicly available. We analyze publicly available strain data from the first Advanced LIGO observing run (O1), obtained through the Gravitational Wave Open Science Center (GWOSC) [16]. Data access and initial processing are performed using the GWpy software package [17].

## Appendix A. Selected Events

The three unmatched coincident excesses not associated with hardware injections identified during the O1 run are presented in this Appendix A (see Figure A1, Figure A2 and Figure A3), illustrating the range of responses of the rank-based detection statistic S(t). While Figure A1 depicts a genuine gravitational-wave signal (GW150914), the remaining two events are identified as instrumental glitches, highlighting the importance of additional data quality veto criteria for large amplitude strain excursions.
